# Supplementary material for: Cytokine‐induced megakaryocytic differentiation is regulated by genome‐wide loss of a uSTAT transcriptional program
Source: EMBO J. 2015 Dec 23;35(6):580–94. doi: 10.15252/embj.201592383 (PMC4801948; doi:10.15252/embj.201592383)
Supplement: Supplementary file 1 — Appendix [file EMBJ-35-580-s001.pdf]

APPENDIX

Appendix Figure Legends

Table S1. Motifs found by de novo motif analysis in the regions bound by uSTAT5 and pSTAT5. The table summarizes all of the motifs found, ranked in order of significance. Each motif is presented with its value of significance (E-value), the most significant match found by TOMTOM (Gupta et al, 2007) in the Jaspar database and the significance value of the match (p-value).

Figure S1. Co-localization of uSTAT5, CTCF and ERG. (A) Venn diagram illustrating overlaps of uSTAT5, CTCF and ERG binding in HPC7 cells. **(B)** Spaced motif analysis (SPAMO; (Whitington et al, 2011)) of uSTAT5 peaks in unstimulated HPC7 cells. **(C)** Snapshot of Szt2/Med8 locus from UCSC genome browser and histogram showing that recruitment of ERG was not affected by STAT5 knock-down.

Figure S2. Genome-wide STAT3 redistribution following cytokine stimulation. (A) Dynamic STAT3 binding regions clustered into 4 groups based on cytokine response to IL-6 and IL-27 as indicated. (B) Motifs identified by *De novo* motif discovery analysis on each cluster of STAT3 binding regions.

Appendix Table S1

Motifs for Stat5 unstimulated		Motifs for Stat5 after Tpo stimulation	
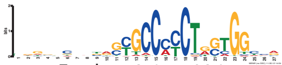<br>E-value=2.6 e-1086	*JASPAR*: CTCF<br>*p*-value= 1.5179e-32	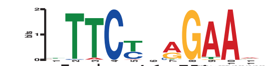<br>E-value=4.6e-751	GAS (*JASPAR*: STAT1 motif)<br>*p*-value= 3.97999e-07
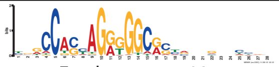<br>E-value=1.4e-1021	*JASPAR*: CTCF<br>*p*-value=6.44191e-34	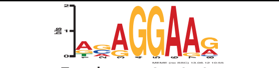<br>E-value=1.2e-070	ETS (*JASPAR*: SPIB motif)<br>*p*-value= 1.72246e-05
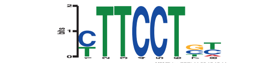<br>E-value=2.5e-343	ETS (*JASPAR*: FEV)<br>*p*-value=1.71532e-05	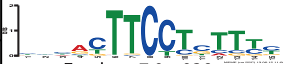<br>E-value=7.0e-039	ETS (*JASPAR*: SPI1 motif)<br>*p*-value= 5.50239e-06
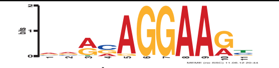<br>E-value=1.2e-336	ETS (*JASPAR*: SPI1)<br>*p*-value=5.64351e-06	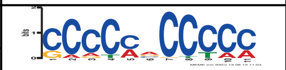<br>E-value=6.4e-012	*JASPAR*: SP1 motif<br>*p*-value= 1.01542e-06
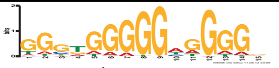<br>E-value=4.6e-061	*JASPAR*: SP1 motif<br>*p*-value=3.12894e-08	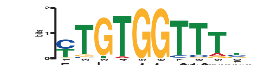<br>E-value=4.4e-010	Runx (*JASPAR*: Runx1 motif)<br>*p*-value= 3.89605e-07
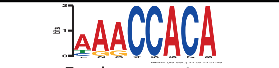<br>E-value=7.4e-057	Runx (*JASPAR*: Runx1 motif)<br>*p*-value=1.0957e-06	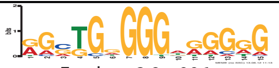<br>E-value=2.9e-006	*JASPAR*: SP1 motif<br>*p*-value= 9.69736e-07
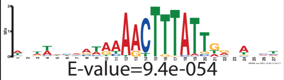<br>E-value=9.4e-054	no matches in *JASPAR*	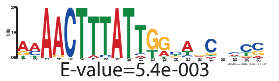<br>E-value=5.4e-003	no matches in *JASPAR*
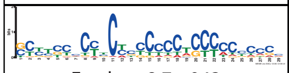<br>E-value=8.7e-043	*JASPAR*: SP1 motif<br>*p*-value = 5.03916e-08		
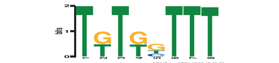<br>E-value=2.1e-034	no matches in *JASPAR*		
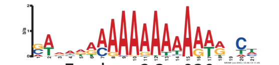<br>E-value=2.3e-030	no matches in *JASPAR*		
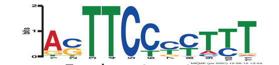<br>E-value=3.1e-020	no matches in *JASPAR*		
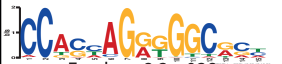<br>E-value=8.2e-028	*JASPAR*: CTCF<br>*p*-value= 4.26873e-21		
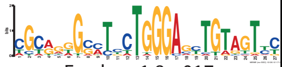<br>E-value=1.8e-017	*JASPAR*: znf143<br>*p*-value=1.55543e-07		
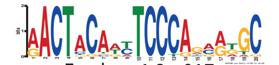<br>E-value=1.8e-017	no matches in *JASPAR*		
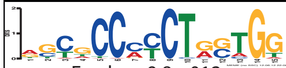<br>E-value=9.8e-012	*JASPAR*: CTCF<br>*p*-value= 4.12067e-21		
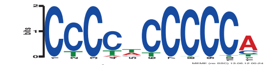<br>E-value=1.1e-009	*JASPAR*: SP1 motif<br>*p*-value= 1.06326e-07		
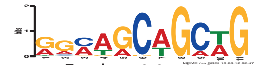<br>E-value=3.3e-013	*JASPAR*: Myf<br>*p*-value=1.94413e-07		
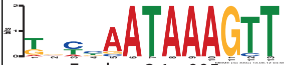<br>E-value=3.1e-005	no matches in *JASPAR*		
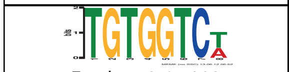<br>E-value=2.6e-003	no matches in *JASPAR*		

Appendix Figure S1

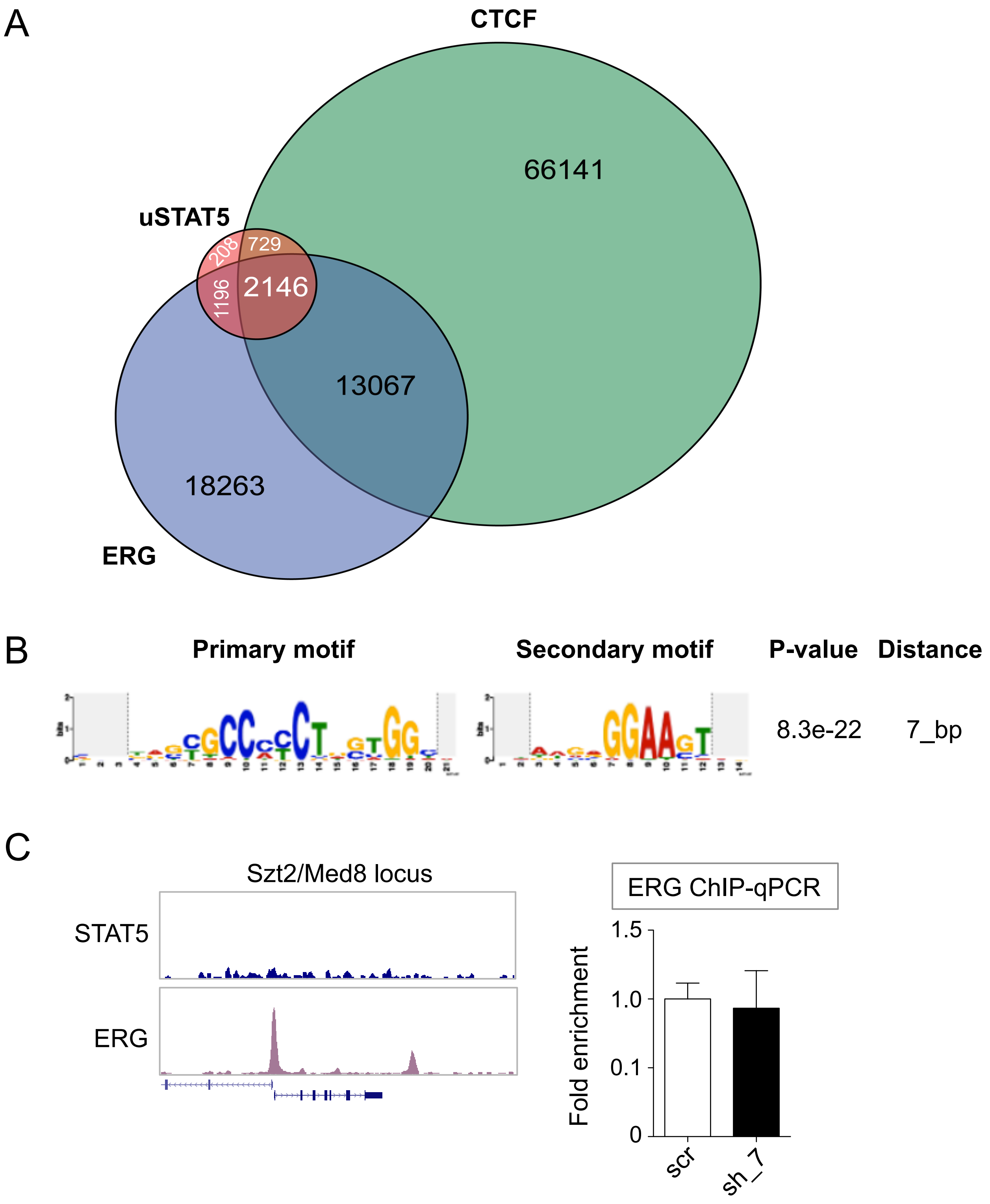

Appendix Figure S2

A

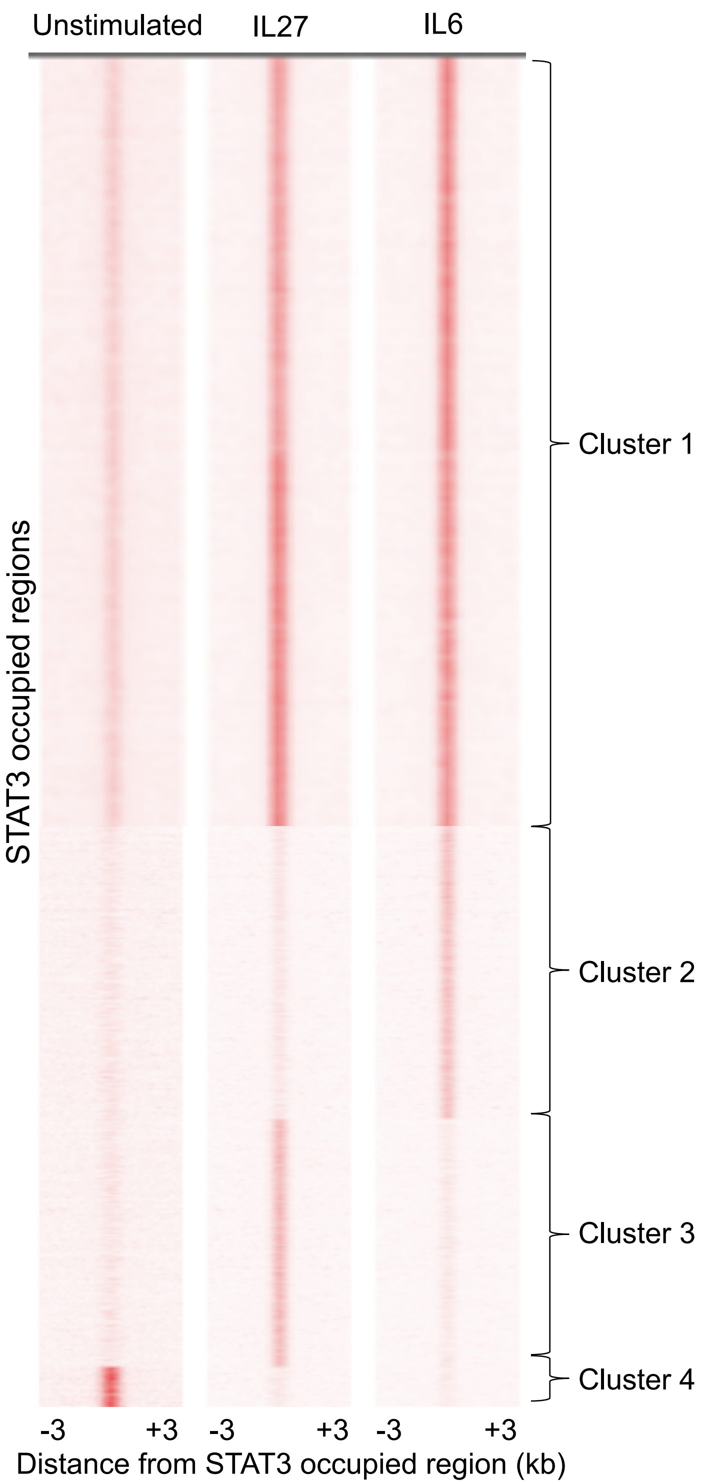

B

TF	p-value	% of peaks with motif	
Jun-AP1 (bZIP)	1e- 4050	44.24%	Cluster 1
Stat3	1e- 2054	38.13%	Cluster 1
ETS	1e- 1292	28.32%	Cluster 1
RUNX1	1e- 549	16.25%	Cluster 1
Atf1	1e- 185	17.14%	Cluster 1
Zfp740	1e- 155	14.48%	Cluster 1
IRF	1e- 126	8.59%	Cluster 1
Sp1	1e- 101	7.27%	Cluster 1
NKX3-1	1e- 97	0.86%	Cluster 1
IRF4	1e- 66	7.21%	Cluster 1
HIF1b(HLH)	1e- 628	28.66%	Cluster 2
Stat3	1e- 372	30.42%	Cluster 2
ETS	1e- 161	13.33%	Cluster 2
Hdx	1e- 107	44.81%	Cluster 2
IRF	1e- 64	21.28%	Cluster 2
FOXC1	1e- 37	7.47%	Cluster 2
RUNX1	1e- 35	12.87%	Cluster 2
Hand1::Tcfe2a	1e- 35	2.99%	Cluster 2
CTCF	1e- 26	1.43%	Cluster 2
Foxj1_2	1e- 22	2.74%	Cluster 2
HIF1b(HLH)	1e- 827	35.52%	Cluster 3
Stat3	1e- 388	44.70%	Cluster 3
RUNX1	1e- 175	15.08%	Cluster 3
PU.1-IRF	1e- 138	21.50%	Cluster 3
IRF	1e- 75	25.17%	Cluster 3
SP1	1e- 61	15.36%	Cluster 3
c-Jun-CRE(bZIP)	1e- 57	12.07%	Cluster 3
ETS	1e- 46	7.63%	Cluster 3
T-box	1e- 44	19.92%	Cluster 3
CTCF	1e- 40	2.14%	Cluster 3
HIF1b(HLH)	1e- 54	18.96%	Cluster 4
NFkB	1e- 36	20.18%	Cluster 4
IRF	1e- 28	7.76%	Cluster 4
MyoG	1e- 26	16.85%	Cluster 4
NFAT:AP1	1e- 20	8.65%	Cluster 4
Irf5	1e- 19	21.73%	Cluster 4
Egr2	1e- 18	9.20%	Cluster 4
STAT5	1e- 18	6.76%	Cluster 4
IRF	1e- 15	7.65%	Cluster 4
Tcf7	1e- 14	4.77%	Cluster 4
