# Supplementary material for: Cytokine‐induced megakaryocytic differentiation is regulated by genome‐wide loss of a uSTAT transcriptional program
Source: EMBO J. 2015 Dec 23;35(6):580–94. doi: 10.15252/embj.201592383 (PMC4801948; doi:10.15252/embj.201592383)
Supplement: Supplementary file 2 — Expanded View Figures PDF [file EMBJ-35-580-s002.pdf]

Expanded View Figures

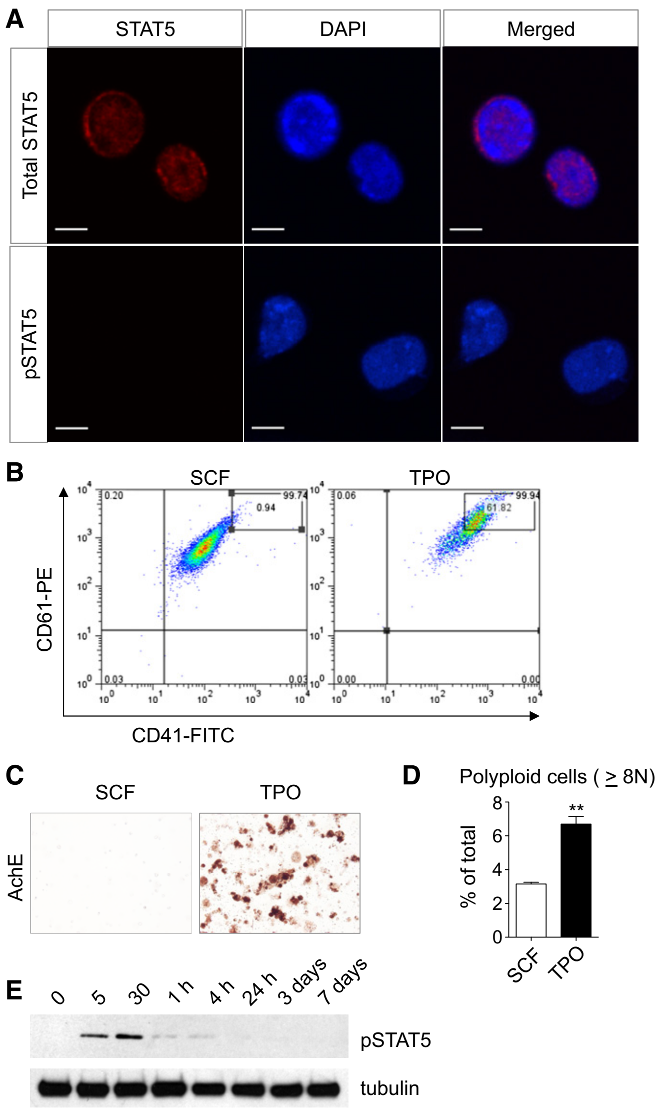

Figure EV1. Nuclear localization of STAT5 proteins in the absence of tyrosine phosphorylation.

A Confocal images showing nuclear STAT5 proteins in primary mouse bone marrow $Lin^{-}Sca\text{-}1^{+}cKit^{+}$ (LSK) cells following serum starvation. Cells were stained with antibodies for total STAT5 or phospho-STAT5 (pY694/699). Fluorescent images were captured by confocal laser scanning microscopy. Scale bars represent 5 μm.

B Flow cytometry plots of HPC7 cells showing an increased frequency of $CD41^{hi}CD61^{hi}$ cell population following TPO stimulation.

C Cytospins of HPC7 cells grown in SCF or TPO were stained for acetylcholinesterase (AchE).

D Histogram showing increased percentage of polyploid HPC7 cells following TPO stimulation. Data represent mean ± SD; $n = 3$; two-tailed Student's *t*-test; $^{**}P < 0.01$.

E Expression of pSTAT5 was examined by Western blots. Total cell lysates were prepared from HPC7 cells at the indicated time points after TPO stimulation.

Figure EV2. Redistribution of STAT5 following TPO stimulation.

A,B Examples of cluster 1 and cluster 2 ChIP-Seq peaks. Raw ChIP-Seq reads were converted to density plots and displayed as custom tracks on the UCSC genome browser.

C Genomic distribution of STAT5 binding sites identified by ChIP-Seq was determined using CEAS (*cis*-regulatory element annotation system) (Shin *et al*, 2009).

D CentriMo analysis of STAT5 ChIP-Seq peak regions (400 bp) showing that the CTCF and ERG motifs are centrally located in uSTAT5 peaks and STAT5 and ERG motifs are centrally located in pSTAT5 peaks.

E Blow-ups of CTCF/uSTAT5 peaks around Mpl and Gp6 locus.

© 2015 The Authors

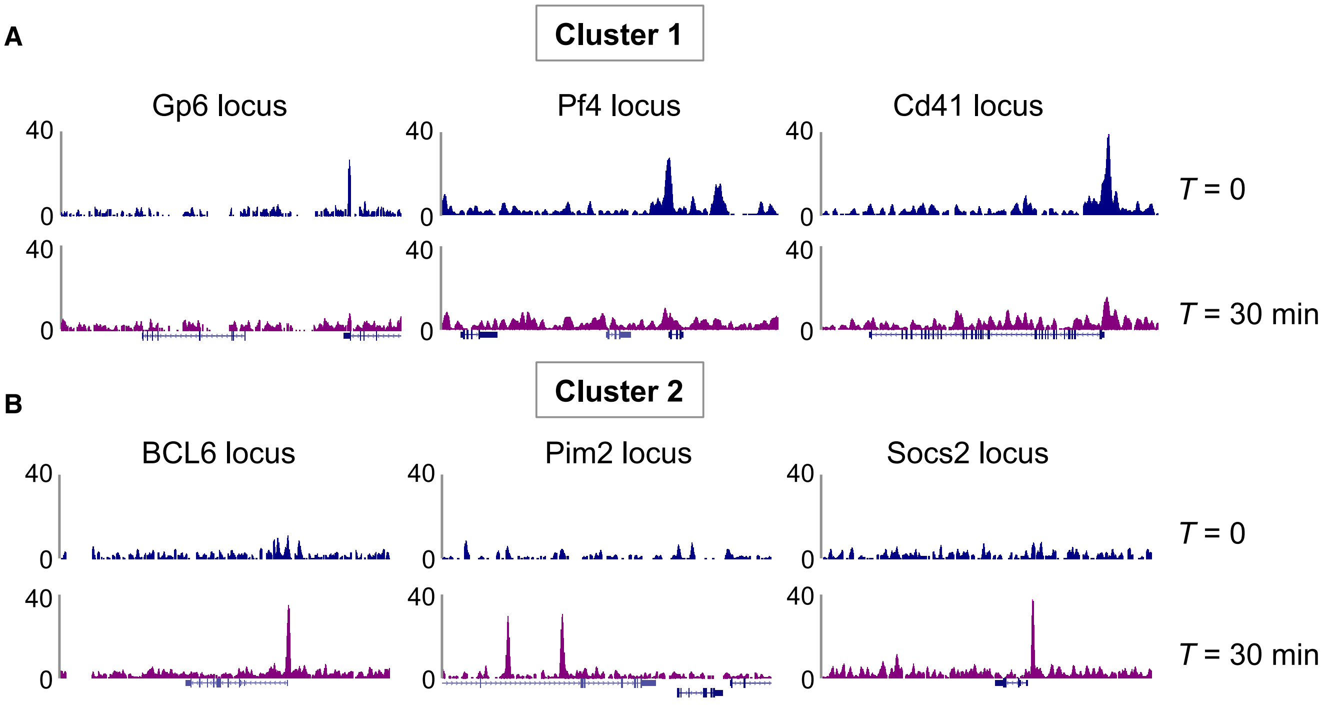

C

	Exon%	Intron%	5'UTR%	3'UTR%	Promoter%	Downstream%	Intergenic%
T = 0	4.1	24.27	0.2	0.64	21.46	2.01	47.32
T = 30	2.42	29.91	0	0.1	7.05	1.01	59.52

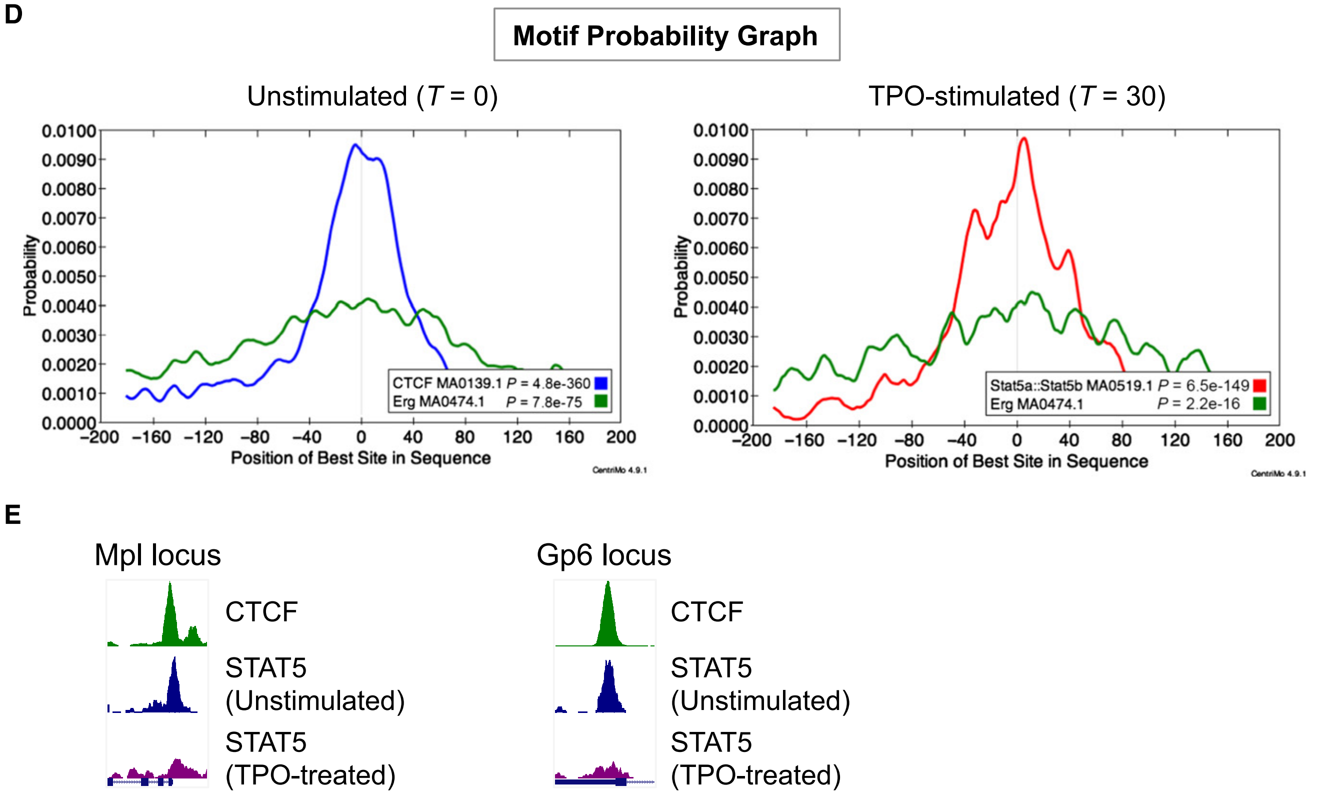

Figure EV2.

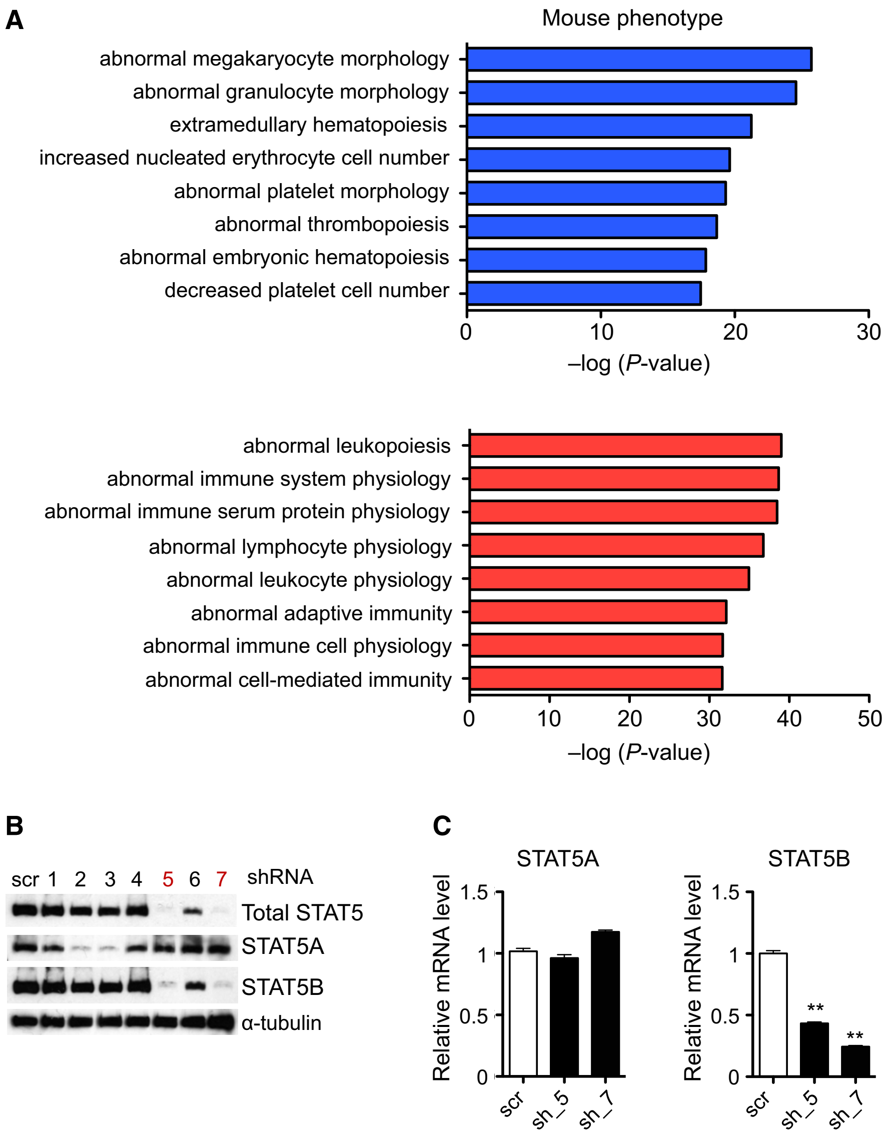

Figure EV3. Analysis of uSTAT5 function.

A GREAT analysis was performed on the genomic regions bound by both uSTAT5 and CTCF (blue) and those bound by pSTAT5 (red).

B, C Knockdown efficiency of 7 independent STAT5 shRNAs (shRNA 1–7) was assessed by Western blot analysis (B) and quantitative RT–PCR (C). Data represent mean ± SD; $n = 3$; two-tailed Student's t-test; $^{**}P < 0.01$.

 © 2015 The Authors

A

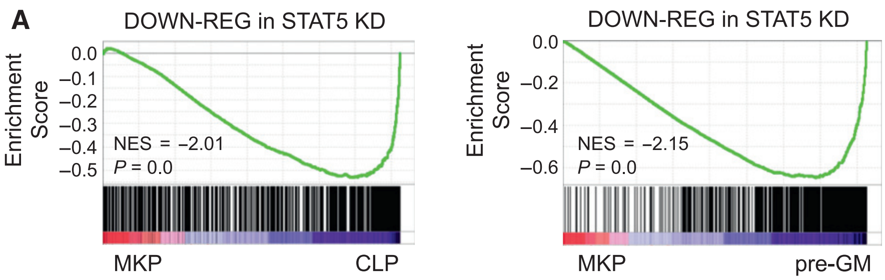

B

Upregulated Genes in STAT5 KD cells (FC > 1.5)

Term	P-value	Fold Enrichment
coagulation	9.20E-09	9.4867884
blood coagulation	9.20E-09	9.4867884
hemostasis	1.09E-08	9.353171662
regulation of body fluid levels	1.65E-08	8.035481963
response to wounding	3.19E-07	3.533097874

C

Downregulated Genes in STAT5 KD cells (FC > 1.5)

Term	P-value	Fold Enrichment
lymphocyte activation	1.94E-11	4.678230105
leukocyte activation	1.06E-10	4.22079334
cell activation	1.71E-09	3.757535535
T-cell activation	5.40E-09	5.312377825
regulation of lymphocyte differentiation	2.64E-07	6.906091172

D

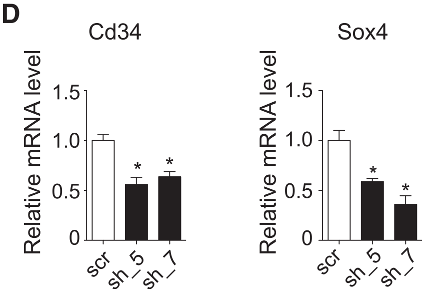

Figure EV4. Transcriptional consequences of uSTAT5 depletion.

A Gene set enrichment analysis (GSEA) of genes downregulated by STAT5 depletion shows a significant enrichment of genes expressed in common lymphoid progenitor (CLP) and in pre-granulocyte and macrophage progenitor (pre-GM).

B, C Gene ontology analysis (using DAVID) of genes upregulated (B) and downregulated (C) by uSTAT5 depletion. *P*-values are raw *P*-values from binominal test.

D Quantitative RT–PCR was performed to validate uSTAT5 target genes. Expression was normalized to 18S rRNA. Histograms represent mean ± SD from three independent experiments. Two-tailed Student's *t*-test; $*P < 0.05$.

Figure EV5. uSTAT5 represses megakaryocytic differentiation of bone marrow LSK cells.

A Schematic diagram of uSTAT5 knockdown in $Lin^{-}Sca\text{-}1^{+}cKit^{+}$ (LSK) cells.

B Flow cytometric analysis showing that uSTAT5 depletion increased the percentage of $CD41^{+}CD61^{+}$ megakaryocytic cells. Bar graph shows mean percentages ± SD of $CD41^{+}CD61^{+}$ cells from three independent experiments.

C Quantitative RT–PCR shows upregulation of megakaryocytic genes following uSTAT5 depletion. Histograms represent means ± SDs from three independent experiments.

D Strategy for assessing the effect of increasing uSTAT5 on megakaryocytic differentiation of LSK cells.

E Flow cytometric analysis showing that the percentage of $CD41^{+}CD61^{+}$ megakaryocytic cells was reduced following the expression of STAT5B Y699F mutant. Histogram shows mean percentages ± SD from three independent experiments.

F Quantitative RT–PCR shows downregulation of megakaryocyte genes following the expression of STAT5B Y699F mutant compared to EV. Transcript levels were normalized to 18S rRNA. Histograms represent mean ± SD from three independent experiments.

Data information: Two-tailed Student's *t*-test; $*P < 0.05$; $**P < 0.01$; $***P < 0.001$.

© 2015 The Authors

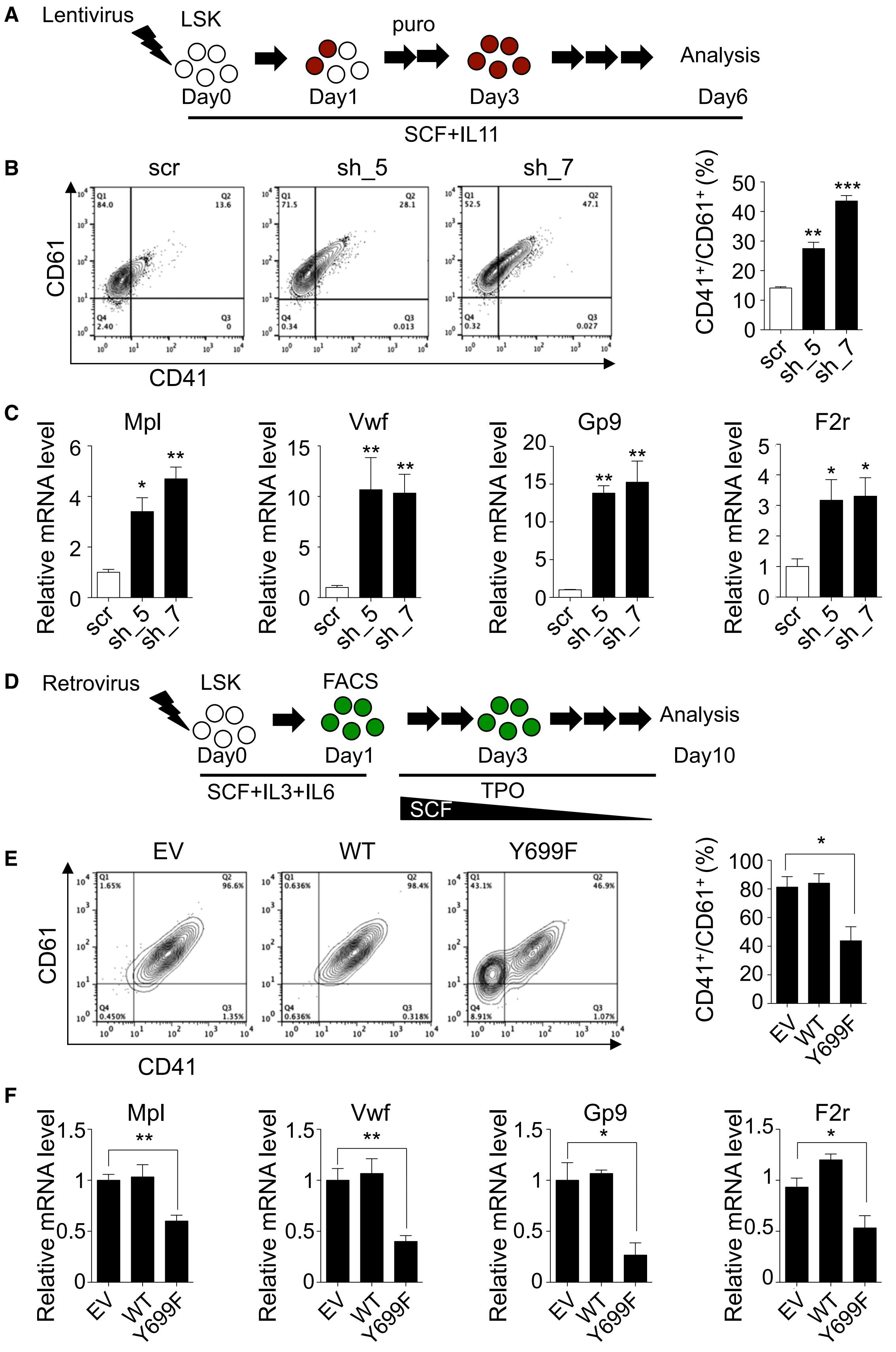

Figure EV5.

© 2015 The Authors
