# Supplementary figures and images for: Cytokine‐induced megakaryocytic differentiation is regulated by genome‐wide loss of a uSTAT transcriptional program
Source: EMBO J. 2015 Dec 23;35(6):580–94. doi: 10.15252/embj.201592383 (PMC4801948; doi:10.15252/embj.201592383)

Source data Figure 1

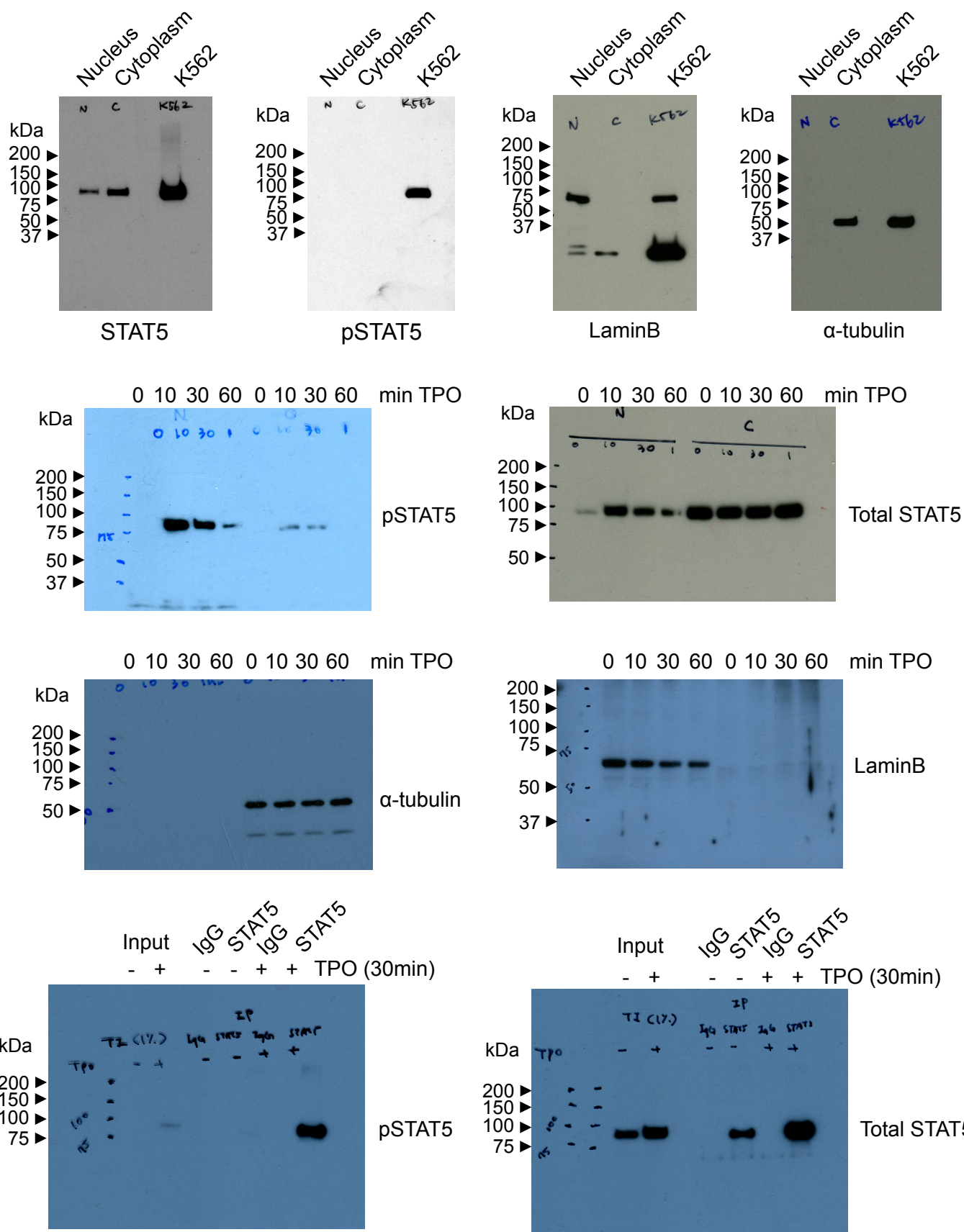

Supplement: Supplementary file 4 — Source Data for Figure 1 [file EMBJ-35-580-s003.pdf]

Source data Figure 2

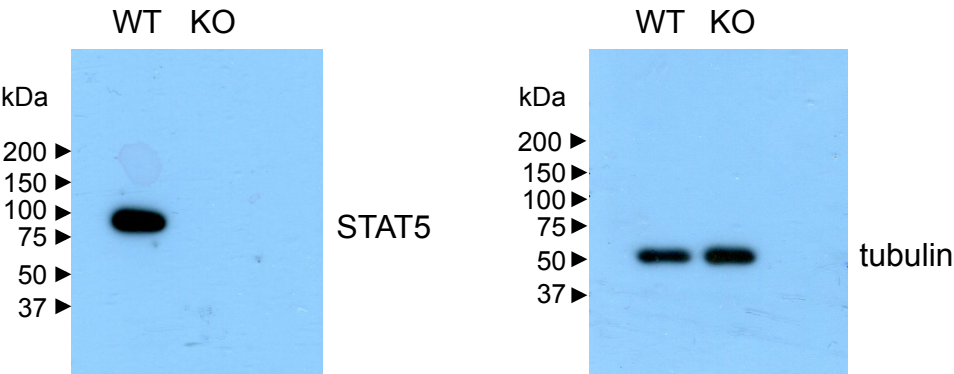

Supplement: Supplementary file 5 — Source Data for Figure 2 [file EMBJ-35-580-s004.pdf]

Source data Figure 3

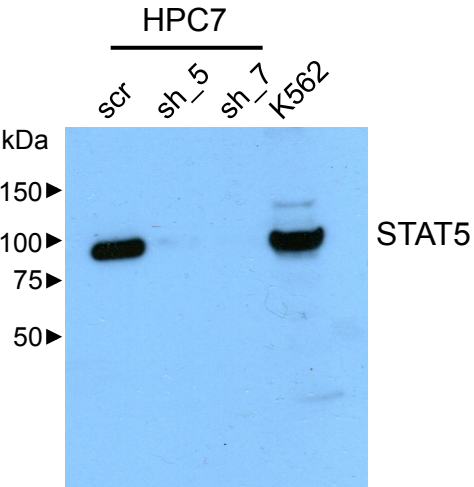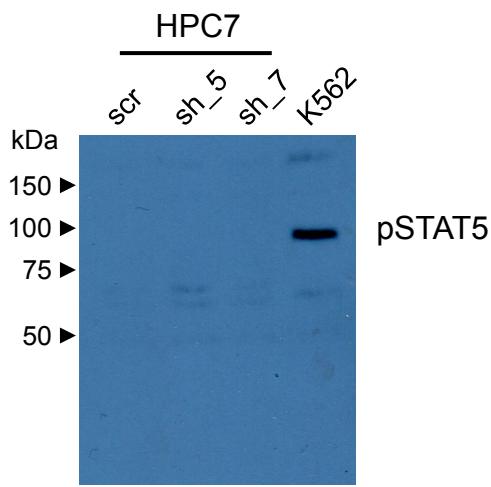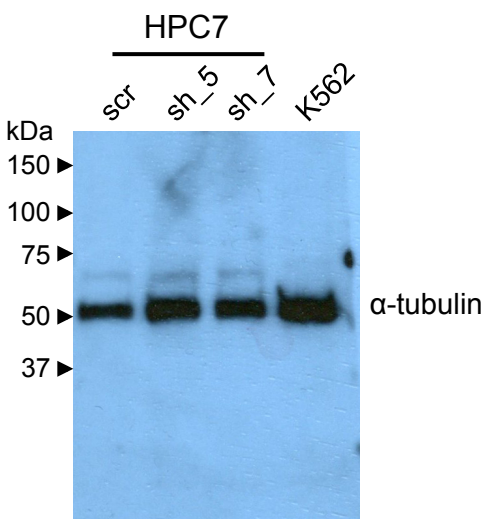

Supplement: Supplementary file 6 — Source Data for Figure 3 [file EMBJ-35-580-s005.pdf]

Source data Figure 4

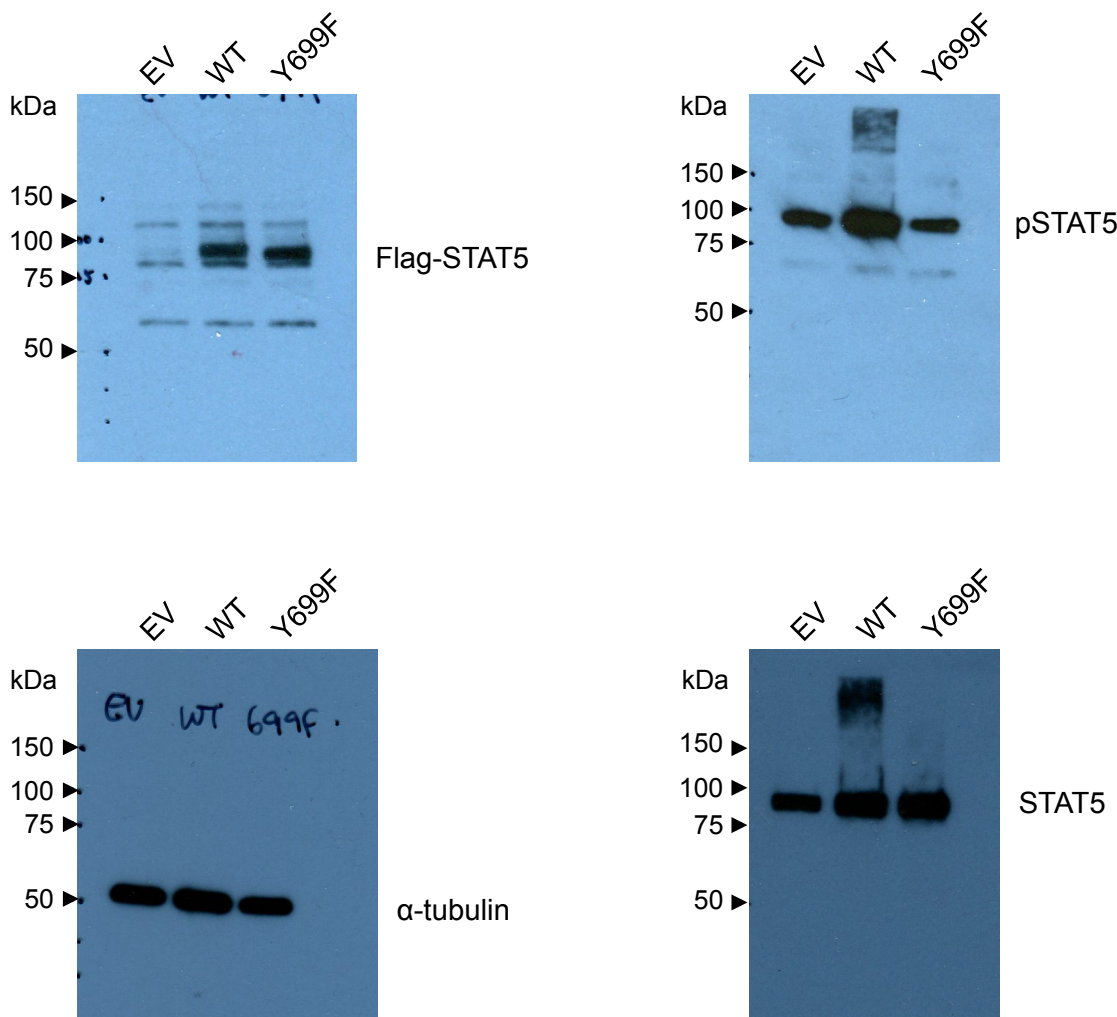

Supplement: Supplementary file 7 — Source Data for Figure 4 [file EMBJ-35-580-s006.pdf]
